# 

**Published:** 1855-12

**Authors:** 


### TRANSACTIONS

OF THE

### EPIDEMIOLOGICAL SOCIETY

### OF LONDON

### FOR THE YEAR 1855.

LONDON:
T. RICHARDS, 37, GREAT QUEEN STREET.

M.DCCC.LVI.

